# Factors affecting HBV DNA suppression in chronic hepatitis B patients treated with tenofovir disoproxil fumarate

**DOI:** 10.12688/f1000research.128116.2

**Published:** 2023-08-24

**Authors:** Darmadi Darmadi, Dharma Lindarto, Jelita Siregar, Tri Widyawati, Muhammad Rusda, Mustafa Mahmud Amin, Fauzi Yusuf, Putri Chairani Eyanoer, Masrul Lubis, Imelda Rey

**Affiliations:** 1 Department of Internal Medicine, Faculty of Medicine, Universitas Sumatera Utara, Medan, North Sumatera, 20155, Indonesia; 2 Philosophy Doctor in Medicine Program, Faculty of Medicine, Universitas Sumatera Utara, Medan, North Sumatera, 20155, Indonesia; 3 Department of Clinical Pathology, Faculty of Medicine, Universitas Sumatera Utara, Medan, North Sumatera, 20155, Indonesia; 4 Department of Pharmacology and Therapeutic, Faculty of Medicine, Universitas Sumatera Utara, Medan, North Sumatera, 20155, Indonesia; 5 Master Program in Tropical Medicine, Faculty of Medicine, Universitas Sumatera Utara, Medan, North Sumatera, 20155, Indonesia; 6 Department of Obstetrics and Gynecology, Faculty of Medicine, Universitas Sumatera Utara, Medan, North Sumatera, 20155, Indonesia; 7 Department of Psychiatry, Faculty of Medicine, Universitas Sumatera Utara, Medan, North Sumatera, 20155, Indonesia; 8 Department of Internal Medicine, Faculty of Medicine, Universitas Syiah Kuala, Banda Aceh, Aceh, 23111, Indonesia; 9 Department of Public Health, Faculty of Medicine, Universitas Sumatera Utara, Medan, North Sumatera, 20155, Indonesia

**Keywords:** CTLA-4, HBV DNA, Hepatitis B, Polymorphism, Tenofovir

## Abstract

**Background: **This study aims to determine the factors affecting HBV DNA suppression in chronic hepatitis B patients with tenofovir disoproxil fumarate (TDF).

**Methods: **A case-control was carried out from October 2021 to August 2022 on 182 chronic hepatitis B patients who had TDF therapy regularly for 24 weeks at H. Adam Malik and USU Hospitals in Medan, Indonesia. The history of the samples was obtained, followed by physical examination, and blood collection. CTLA-4 polymorphism examination was carried out using real-time PCR, while the serum CTLA-4 levels were assessed with ELISA.

**Results: **The CTLA-4 -1661G>A polymorphism, genotype GG+AG, increased 1.52 times risk of not achieving HBV DNA suppression to TDF compared to genotype AA (p=0.041). High CTLA-4 levels increased 2.28 times risk, high HBV DNA levels increased 2.09 times risk, low ALT levels increased 1.95 times risk of not achieving HBV DNA suppression (p= 0.009, 0.026, 0.036, respectively). There was no relationship between gender, age, ethnicity, obesity, baseline AST, HBeAg, genotype, liver fibrosis and HBV DNA suppression after 24 weeks of treatment (p>0.05).

**Conclusions: **The levels of CTLA-4, HBV DNA, ALT, and CTLA-4 -1661G>A polymorphism have a potential relationship with the suppression of HBV DNA in chronic hepatitis B patients with TDF.

## Introduction

Hepatitis B (HBV) is a health problem affecting several countries in the world, including Indonesia. A national survey in 2013 showed the proportion of positive Hepatitis B surface antigen (HBsAg) was 7.1% ( Indonesia Health Ministry, 2013). The ideal endpoint is HBsAg clearance, a satisfactory endpoint is HBeAg seroconversion, and the next most desirable endpoint is persistent inhibition of HBV DNA replication. In practical experience, the loss of HBsAg as an ideal therapeutic endpoint is often difficult to achieve, hence, treatment is only focused on the satisfactory and desirable endpoints. Tenofovir disoproxil fumarate (TDF) is one of the first-line drugs used for the treatment of chronic hepatitis B ( INA ASL, 2017). Several studies have explored its antiviral mechanism, but the immunomodulatory mechanism is still being studied. TDF has been reported to have immunomodulatory activity, and it can reduce Treg cell levels in chronic hepatitis B patients ( Yu *et al.*, 2013). It can also reduce the release of proinflammatory cytokines, such as IL-8 and CCL3 ( Melchjorsen *et al*., 2011). Treatment with NA enhances specific T-cell function and anergy by regulating CTLA-4 ( Thimme and Dandri, 2013). Previous studies showed that long-term NA therapy can increase peripheral T cell levels to enhance antiviral response ( Zheng *et al*., 2012). Furthermore, their activation is strongly determined by genetic factors, such as CTLA-4 ( Schurich *et al.*, 2011; Zheng *et al.*, 2012).

CTLA-4 levels in the body are influenced by allele variations in the CTLA-4 gene, which is located on chromosome 2q33.3 and consists of 4 exons and 3 introns ( Schneider and Rudd, 2014). Chen's study in China revealed that the CTLA-4 +49G>A gene polymorphism of the GG genotype increased the risk of developing liver cirrhosis and HCC, while CTLA-4 -318 T>C of CC genotype increased the risk of persistent hepatitis B virus infection ( Chen *et al.*, 2014). Wang stated that chronic HBV patients with CTLA-4 +49A>G allele G and genotype GG were significantly more at risk of developing HCC in ethnic Chinese ( Wang *et al.*, 2018).

These studies have focused on the association of CTLA-4 polymorphism with the persistence and progression of chronic hepatitis B infection, but to our knowledge, no studies have explored its relationship with HBV DNA suppression. Therefore, this study aims to determine the association of the CTLA-4-1661G>A gene polymorphism and other factors, such as age, gender, ethnicity, obesity, baseline AST, baseline ALT, baseline HBV DNA, HBeAg, genotype, degree of fibrosis, and CTLA-4 levels with HBV DNA suppression in chronic hepatitis B patients treated with TDF.

## Methods

### Study design

A case-control study was carried out from October 2021 to August 2022 on 182 chronic hepatitis B patients who had already done 24 weeks therapy with TDF at H. Adam Malik and USU Hospitals in Medan, Indonesia. Medication adherence was observed by self-reporting diary. The case group was patients who did not achieve HBV DNA suppression after 24 weeks of TDF treatment. The control group was patients who achieved HBV DNA suppression after 24 weeks of TDF treatment. The research samples were taken using consecutive sampling technique. All subjects who came and met the study criteria were included consecutively in the case or control group until the number of samples was fulfilled.

### Ethical considerations

Ethical approval was obtained from the Ethics Review Board of Universitas Sumatera Utara on October 18 ^th^, 2021 (Ethical clearance number: 1028/KEP/USU/2021). Written informed consent was obtained from participants before enrollment in this study ( Darmadi, 2022c).

### Sample

To determine the sample size, use the following formula:

$$\frac{{\left(Z_{\alpha }\sqrt{2\mathrm{pq}}+Z_{\beta }\sqrt{p_{1}q_{1}+p_{2}q_{2}}\right)}^{2}}{\left(p_{1}-p_{2}\right)}$$

Zα = 1.96, Zβ = 1.282, p1 (proportion of patients with the GG+GA genotypes of CTLA-4 -1661 G>A polymorphism who did not achieve HBV DNA suppression) = 0.57, p2 (Proportion of patients with the GG+GA genotypes of CTLA-4-1661G>A polymorphism who achieve HBV DNA suppression) = 0.23 ( Wang *et al.*, 2007). The minimum sample size for each group was 91 subjects. The minimum total sample size was 182 subjects.

Inclusion criteria in this study were chronic hepatitis B patients who were on TDF therapy routinely for 24 weeks, aged > 18 years, were cooperative and willing to participate by signing an informed consent form. Patients who received Peg-IFN or other NA therapies, consumed immunomodulatory/immunosuppressant drugs for the past 1 month, have liver cirrhosis, hepatocellular carcinoma, systemic diseases, such as HIV, chronic kidney disease, autoimmune, malignancy, and pregnancy were excluded from the study.

### Procedure

The selected patients were interviewed using a form to obtain age, ethnicity, and comorbid diseases, after which a physical examination was carried out by a hepatologist on duty.

Abdominal ultrasound was performed to evaluate signs of liver cirrhosis, such as reduced liver size, irregular surface, heterogeneous parenchyma, ascites, splenomegaly, as well as signs of hepatocellular carcinoma. Transient elastography with fibroscan is a non-invasive method to evaluate the degree of fibrosis. Furthermore, its degree in hepatitis B consists of 4 categories, namely F0-F1/normal – mild when < 6.0 kPa, F2/significant fibrosis > 6 kPa, F3/severe fibrosis > 9 kPa, F4/liver cirrhosis > 12 kPa ( Bonder and Afdhal, 2014). ALT, HBV DNA, HBeAg, anti-HBe, and CTLA-4 data were collected before starting treatment. Patients were examined for ALT, HBV DNA, HBeAg, and anti-HBe after 24 weeks of TDF treatment.

The determination of serum ALT levels was carried out using standard biochemical tests with an Olympics analyzer AU400 (Hamburg, Germany). The HBeAg and anti-HBe antibody levels were examined using a commercial enzyme-linked immunosorbent assay (ELISA) kit (Roche ELISA kit, Germany). The HBV DNA viral load was assessed with the COBAS TaqMan HBV Test, version 2.0 (Roche Diagnostics, Tokyo, Japan), with a measurement ratio of 2.1 – 9 log copies/mL. We used the kits according the manufacturer’s instructions.

### DNA isolation

DNA extraction was carried out with Genomic DNA Mini Kit (Geneaid Biotech Ltd., New Taipei City, Taiwan), Cat GB100, Lot. FG02009) using whole blood approximately 5 ml. All the procedure for CTLA-5-1661 G>A were performed in Prodia Laboratorium, Medam, Indonesia. All the procedure under the working procedure according to the kit.

The Primer & Probe used in this study were TaqMan SNP Genotyping Assay CTLA-4 -1661 G>A (Applied Biosystems, CA, USA) cat:44033111, Lot. 00905960. Genotyping of the CTLA-4-1661G>A gene was performed with a forward primer: 5′-CTAAGAGCATCCGCTTGCACCT-3′ and a reverse primer: 5′ TTGGTGTGATGCACAGAAGCCTTTT-3′.

Amplification was performed with a C1000 thermal cycler CFX96 real-time system (BioRad, CA, USA) using the following standard protocol. The reaction mixture for RT-PCR used the TaqMan GTXpress master mix (2x) reagent with a total volume of 25 ul TaqMan GTXpress Master Mix (2x), added 1.25 ul 20x working stock SNP genotyping assay, 6.25 ul ddH20, and 5 ul DNA template with a concentration of 1-10ng/well. Amplification was performed using a CFX 96 touch ^TM^ RT-PCR (Biorad) with the following standard protocol: 20 seconds of enzyme activation at 95°C, followed by 40 amplification cycles consisting of 15 seconds of denaturation at 95°C, and 1 minute of annealing/extension at 60 C.

### Plasma CTLA-4 levels

The measurement of serum CTLA-4 levels was performed with the ELISA method (Quantikine *(R)* ELISA with catalog number HSCT40, Human CTLA-4 Immunoassay R&D System Inc., Minneapolis, USA). All the materials we need, namely human CTLA-4 capture antibody (lyophilized), human CTLA-4 detector antibody 10x, human CTLA-4 lyophilized recombinant protein, antibody diluent 4BI, cell extraction buffer PTR 5X, cell extraction buffer enhancer solution 50X, stop solution, TMB development solution, wash buffer PT 10X, and sample diluent NS. All the items store in 4 C.

After preparing all reagents, standards, and samples (serum), open the seal plate, put it back in the foil pouch, and store it at 4°C. Then add 50 μL of sample or standard to each well. After that, add 50 μL Antibody Cocktail to each well.

The plate was closed with a seal, then incubated for 1 hour at room temperature on a shaker at 400 rpm. Afterward, wash each well with 3 x 350 μL 1X Wash Buffer. In the last washing, turn the plate over and dry, then add 100 μL TMB Development Solution to each well and incubate for 10 minutes in the dark on a shaker at 400 rpm. Adding the Stop Solution will change the color from blue to yellow and increase the signal intensity by about 3X. To avoid signal saturation, proceed to the next step before the high concentration of standard reaches a blue color OD 600 equals 1.0. Given the variability in laboratory environmental conditions, the optimal incubation time can vary between 5 and 20 minutes.

In the final stage, add 100 μL Stop Solution to each well. It alternates the plate so that it is homogeneous. Then read at a wavelength of 450 nm.

### Data analysis

Data analysis was carried out using the chi-square test and binomial logistic regression. Hardy Weinberg equilibrium was tested by chi square analysis. The significance of the statistical test results was determined based on the p-value <0.05 using SPSS-22 Statistics for Windows (IBM Corp, Armonk, NY, USA). Cut off used in age classification, AST level, ALT levels, HBV DNA based on the mean of the study sample was 44 years, 114 IU/L, 120 IU/L, and 6.31 log 10 IU/mL. Cut off for CTLA-4 classification based on the results of the ROC curve analysis.

## Results

**Figure 1.**
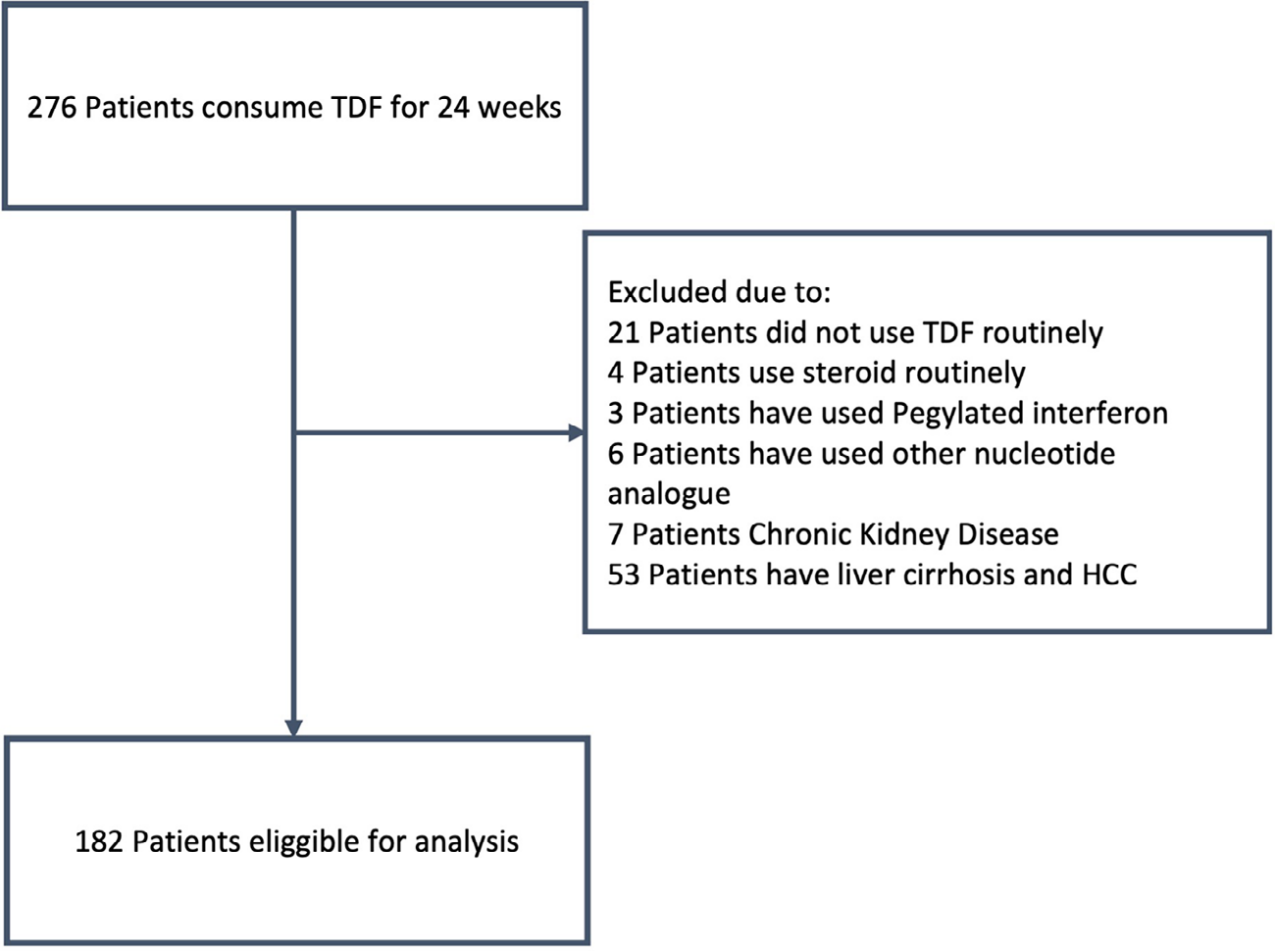
The procedure to obtain sample.

Figure 1 shows the participant flow. Table 1 shows the demographic and laboratory characteristics.

**Table 1.** Relationship between demographic and laboratory characteristics with HBV DNA suppression.

Variable	HBV DNA suppression		P	OR (95% CI)
	No	Yes		
Gender				
Male	54 (48.2)	58 (51.8)	0.542	0.91 (0.68-1.22)
Female	37 (52.9)	33 (47.1)		
Age				
Old	51 (53.7)	44 (46.3)	0.299	1.17 (0.87-1.57)
Young	40 (46)	47 (54)		
Ethnic group				
Batak	58 (51.3)	55 (48.7)	0.647	1.07 (0.79-1.46)
Non-Batak	33 (47.8)	36 (52.2)		
Obesity				
Yes	29 (46)	34 (54)	0.436	0.88 (0.64-1.21)
No	62 (52.1)	57 (47.9)		
AST baseline				
Low	53 (54.1)	45 (45.9)	0.234	1.2 (0.89-1.61)
High	38 (45.2)	46 (54.8)		
ALT baseline				
Low	56 (57.1)	42 (42.9)	0.037 *	1.37 (1.01-1.86)
High	35 (41.7)	49 (58.3)		
DNA VHB *baseline*				
High	49 (59.8)	33 (40.2)	0.017 *	1.42 (1.06-1.9)
Low	42 (42)	58 (58)		
HBeAg				
Positive	43 (53.1)	38 (46.9)	0.456	1.12 (0.84-1.5)
Negative	48 (47.5)	53 (52.5)		
Genotype				
C	50 (48.1)	54 (51.9)	0.549	0.92 (0.68-1.22)
B	41 (52.6)	37 (47.4)		
Liver fibrosis				
F0-F2	76 (51.7)	71 (48.3)	0.347	1.21 (0.8-1.82)
F3	15 (42.9)	20 (57.1)		
CTLA-4 baseline				
High	55 (61.8)	34 (38.2)	0.002 *	1.6 (1.18-2.16)
Low	36 (38.7)	57 (61.3)		

* p<0.05, HBV: hepatitis B virus, AST: aspartate aminotransferase, ALT: alanine aminotransferase, CTLA-4: cytotoxic T-lymphocyte associated protein 4, OR: odds ratio.

There was a significant relationship between low baseline ALT, high baseline HBV DNA, and high CTLA-4 levels with not achieving HBV DNA suppression (p<0.05) ( Darmadi, 2022a, 2022b). Hepatitis B patients with low baseline ALT are 1.37 times more at risk of not achieving HBV DNA suppression (p=0.037), while others with high HBV DNA are 1.42 times more at risk (p=0.017). Furthermore, people with high CTLA-4 levels are 1.6 times more at risk of not achieving suppression compared to others with low levels (p=0.002). The results showed that there was no association between gender, age, ethnicity, obesity, baseline AST, HBeAg, genotype, liver fibrosis, and HBV DNA suppression (p>0.05), as shown in Table 1.

**Figure 2.**
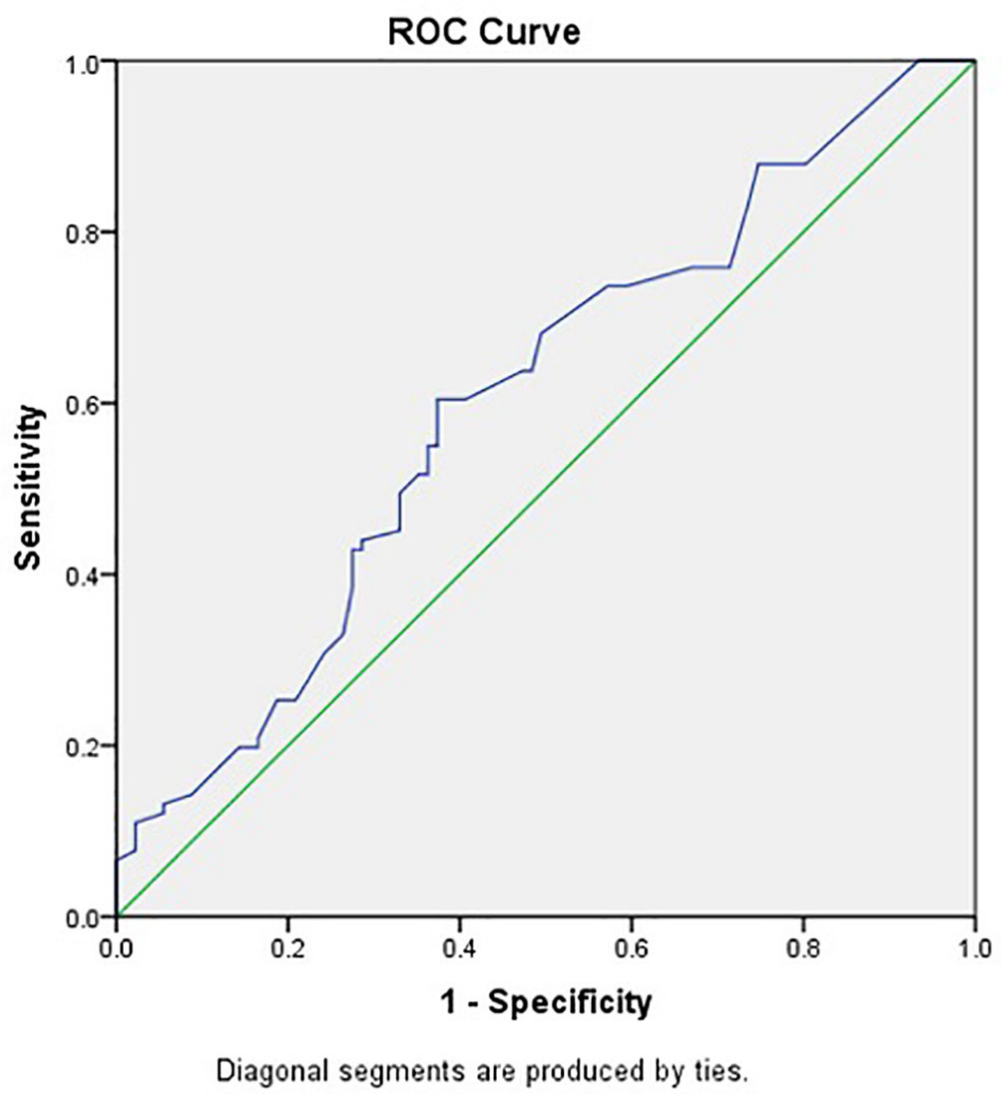
CTLA-4 Receiver Operating Characteristic curve in predicting HBV DNA suppression by TDF.

CTLA-4 levels were significantly higher in chronic hepatitis B patients without HBV DNA suppression compared to others (p=0.016), as shown in Table 2. This study used a value of 52.2 pg/mL as the cut-off to distinguish high and low CTLA-4 levels based on the results of ROC analysis with an area under the curve of 0.603 (p=0.016), as shown in Figure 2.

**Table 2.** Differences in CTLA-4 levels between chronic hepatitis B patients who achieve and do not achieve HBV DNA suppression.

Variable	HBV DNA suppression		P
	No N (%)	Yes N (%)	
CTLA-4 (pg/mL)	67.2 (39.6 – 264)	45.9 (36 – 198)	0.016 *

* p<0.05, HBV: hepatitis B virus, CTLA-4: cytotoxic T-lymphocyte associated protein 4.

The accuracy of CTLA-4 in predicting HBV DNA suppression in chronic hepatitis B patients with TDF is presented in Table 3.

**Table 3.** Diagnostic accuracy of CTLA-4 in predicting HBV DNA suppression in chronic hepatitis B patients with TDF.

*Cut off*	Sens	Spec	PPV	NPV	PLR	NLR	Accuracy
≥52.2 pg/mL	60.4%	62.6%	61.8%	61.3%	1.61	0.63	61.5%

Sens: sensitivity, Spec: specificity, PPV: positive predictive value, NPV: negative predictive value, PLR: positive likelihood ratio, NLR: negative likelihood ratio, HBV: hepatitis B virus, CTLA-4: cytotoxic T-lymphocyte associated protein 4.

CTLA-4 levels of 52.2 pg/mL were able to predict the suppression of HBV DNA by TDF with a sensitivity and specificity of 60.4% and 62.6%, respectively, as shown in Table 3.

The genotype frequency of the -1661G>>A of CTLA-4 polymorphism in the case group (p= 0.987) and control group (p= 0.879) in Hardy Weinberg equilibrium (p>0.05) as shown in Table 4.

**Table 4.** Hardy Weinberg Equilibrium.

CTLA-4-1661G>A polymorphism	HBV DNA suppression					
	No			Yes		
	O	E	X2, p	O	E	X2, p
GG	19	19,38	0,0263	12	10,9	0,2578
AG	46	45,23	p=0,987	39	41,19	p=0,879
AA	26	26,38		40	38,9	

O: observed, E: expected, HBV: hepatitis B virus, CTLA-4: cytotoxic T-lymphocyte associated protein 4.

There was a significant relationship between the CTLA-4-1661G>A polymorphism and HBV DNA suppression. The GG+AG genotype increased 1.42 times risk of not achieving HBV DNA suppression compared to the AA genotype (p=0.031). While there was no relationship between the GG genotype and HBV DNA suppression compared to the AG+AA genotype. Furthermore, patients with the G allele were 1.27 times more at risk compared to others with the A allele (p=0.025), as shown in Table 5.

**Table 5.** Relationship between CTLA-4-1661G>A polymorphism and HBV DNA suppression.

Polymorphism CTLA-4-1661G>A	HBV DNA suppression		p	OR (95% CI)
	No n (%)	Yes n (%)		
GG	19 (61.3)	12 (38.7)	0.077	NA
AG	46 (54.1)	39 (45.9)		
AA	26 (39.4)	40 (60.6)		
GG+AG	65 (56)	51 (44)	0.031 *	1.42 (1.01-2)
AA	26 (39.4)	40 (60.6)		
GG	19 (61.3)	12 (38.7)	0.168	1.29 (0.93-1.78)
AG+AA	72 (47.7)	79 (52.3)		
Alel G	84 (57.1)	63 (42.9)	0.025 *	1.27 (1.03 – 1.55)
Alel A	98 (45.2)	119 (54.8)		

* p<0,05.

The multivariate analysis showed that the levels of CTLA-4, HBV DNA, ALT, and CTLA-4 -1661G>A polymorphisms are associated with the suppression of HBV DNA. Furthermore, high CTLA-4 levels significantly increased the risk of not achieving suppression by 2.28 times (p=0.009). High HBV DNA significantly increased the risk by 2.09 times (p=0.026), while patients with low baseline ALT are 1.95 times more at risk (p=0.036). People with polymorphism CTLA-4 -1661G>A genotype GG+AG are 1.52 times more at risk of not achieving HBV DNA suppression (p=0.041), as shown in Table 6. The value of Nagelkerke R Square was 0.584, which indicates that the ability of the variable levels of CTLA-4, HBV DNA, ALT, and CTLA-4-1661G>A polymorphism in explaining HBV DNA suppression is 0.584 or 58.4%. There are 100% – 58.4% = 57.4% other factors outside the model that explain the dependent variable.

**Table 6.** Multivariate analysis of factors associated with HBV DNA suppression.

Variable	B	p	OR (95% CI)
High CTLA-4 levels	2.055	0.009 *	2.28 (1.23-4.22)
High HBV DNA	1.722	0.026 *	2.09 (1.08-3.75)
Low ALT	1.599	0.036 *	1.95 (1.04-3.67)
CTLA-4-1661G>A polymorphism	1.204	0.041 *	1.52 (1.02-3.55)
GG+AG genotype			
Constant	-7.815		

* p<0,05, HBV: hepatitis B virus, ALT: alanine aminotransferase, CTLA-4: cytotoxic T-lymphocyte associated protein 4.

Based on the B values obtained from the calculation above, the equation model formed was: Ln P/1-P = -7.815 + 2.055 high CTLA-4 + 1.722 high HBV DNA + 1.599 low ALT + 1.204 CTLA-4 polymorphism - 1661G>A. The derivative formula of the equation can also be used, namely Probability = exp (-7.815 + 2.055 high CTLA-4 + 1.722 high HBV DNA + 1.599 low ALT + 1.204 CTLA-4 polymorphism -1661G>A) /1 + exp (-7.815 + 2.055 high CTLA-4 + 1.722 high HBV DNA + 1.599 low ALT + 1.204 CTLA-4 polymorphism -1661G>A).

There were significant differences in the serum CTLA-4 levels between the genotypes of CTLA-4-1661G>A polymorphism. Furthermore, CTLA-4 levels were significantly higher in hepatitis B patients with genotypes GG and AG compared to others with AA (p<0.001). It was also significantly higher in people with the G allele compared to the A allele (p<0.001), as shown in Table 7.

**Table 7.** Differences in Serum CTLA-4 Levels between GG, AG, AA Genotypes and G, A Alleles from the CTLA-4-1661G>A Polymorphism.

CTLA-4-1661G>A Polymorphism	CTLA-4 levels (pg/mL) median (min – max)	p
GG genotype	106.8 (36 – 264) ^#^ ^!^	<0.001 *
AG genotype	67.2 (37 – 228.9) !	
AA genotype	43.6 (37 – 169.2)	
G allele	95.4 (36 – 264)	<0.001 *
A allele	45.9 (37 – 228.9)	

HBV: hepatitis B virus, CTLA-4: cytotoxic T-lymphocyte associated protein 4.

* p<0.05.

^#^ significant compared to the AG genotype.

^!^ significant compared to the AA genotype.

## Discussion

Chronic hepatitis B is a health problem affecting several countries in the world, including Indonesia and it has high morbidity and mortality due to its complications. Furthermore, the importance of management in patients with the disease is to achieve treatment endpoints and to prevent its complications. Tenofovir disoproxil fumarate (TDF) is one of the NA classes of drugs used as the first line of treatment ( INA ASL, 2017). It also has a high antiviral potency as well as 0% resistance after 5 years of use ( De Clercq *et al.*, 2010). Apart from its antiviral activity, TDF can also increase the levels of T cells in the periphery. The activity of these cells is influenced by CTLA4, which can transmit inhibitory signals to them after binding to CD80 and CD86 in APC ( Schurich *et al.*, 2011; Zheng *et al.*, 2012). CTLA-4 level can be influenced by the CTLA-4 gene polymorphism ( Corvalan, Carrasco and Saavendra, 2012), but no study has explored its relationship with response to hepatitis B treatment. Therefore, this study was carried out to analyze the factors influencing HBV DNA suppression, including the CTLA-4-1661G>A polymorphism in chronic HBV patients with TDF.

Men are more prone to chronic hepatitis B infection due to immune, hormonal, and risk factors ( Klein, 2012). Kosinska revealed that male murine models were associated with a worse HBV-specific T-cell response ( Kosinska *et al.*, 2017). The effect of gender on treatment response is controversial, but several studies reported that there is a relationship between them ( Belci *et al.*, 2016; Villa *et al.*, 2011). The incidence of NAFLD and its severity is higher in women, especially postmenopausal compared to men ( Villa *et al.*, 2011). This indicates that NAFLD has an effect on the progression of fibrosis from mild to moderate or severe, which can cause a decrease in the response to antiviral therapy ( Fierbinţeanu-Braticevici *et al.*, 2010; Villa *et al.*, 2011). Meanwhile, other studies stated that women aged <50 years have a better virological response to therapy, but there was no difference in response between men and women >50 years ( Belci *et al.*, 2016). Our study showed that there was no relationship between gender and response to chronic hepatitis B treatment (p=0.542), but it does not stratify the association between the two variables based on age group. Previous studies revealed that gender has no effect on HBV DNA suppression in chronic hepatitis B patients with TDF ( Kau *et al.*, 2008; Lim *et al.*, 2007).

Kennedy stated that the age group with the worse T-cell response to HBV was children ( Kennedy *et al.*, 2012). Another study on adults with chronic HBV showed that patients < 36 years had better immune and virological responses compared to others ( Bonino *et al.*, 2007). This study also found that older people did not achieve HBV DNA suppression, while younger people had more probability, but there was no statistically significant difference (p=0.299). Other studies showed that age and treatment response have no association with NA ( Lim *et al.*, 2007). Tan reported that epidemiological factors, such as age and sex do have an effect on HBV-specific T cell counts. The result also showed that response to treatment has no association with NA ( Tan *et al.*, 2008).

This study revealed that there was no association between ethnicity and HBV DNA suppression in chronic hepatitis B patients with TDF (p=0.647). This is in line with previous studies that ethnicity/race has no relationship with virological response to NA or Peg-IFN ( Kau *et al.*, 2008; Lim *et al.*, 2007). Asian people have been found to be infected with chronic hepatitis B through vertical transmission ( Tan *et al.*, 2008). Ethnicity and route of transmission do not affect the response to hepatitis B treatment for NA or Peg-IFN ( Tan *et al.*, 2008; Lin and Kao, 2013).

Animal studies showed that obese mice have lower levels of antibodies and T cells compared to non-obese mice ( Liu *et al.*, 2017). Studies from South Korea also reported that memory T-cell response function can be affected by obesity ( Park *et al.*, 2014). Furthermore, there was an increase in leptin, which can induce systemic inflammation and B cells in obese patients. Leptin is produced by fat cells and can modulate adaptive and innate immune responses. The effect of obesity on the immune system has been reported by several animal studies, while its effect on response to hepatitis B treatment has not been explored ( Frasca *et al.*, 2013). This finding is consistent with this study, where obesity had no association with HBV DNA suppression in chronic hepatitis B patients with TDF (p=0.436).

ALT is an enzyme produced by hepatocytes and a highly specific biomarker for hepatocellular injury ( Hall and Cash, 2012). Patients’ ALT level is an important factor in the initiation of chronic hepatitis B therapy. Increased levels of the enzyme indicate high immune-mediated inflammation to eliminate HBV-infected hepatocytes. It has also become an important aspect in predicting the virological response of chronic hepatitis B patients in terms of HBV DNA suppression and HBeAg seroconversion ( Lok, 2019). In the GLOBE study, ALT levels > 2x the upper limit of normal were predictors of virological response to NA treatment ( Zeuzem *et al.*, 2009). A 4-year case-management study of HBeAg positive patients showed that 78% of people with ALT >10 upper limit of normal had HBeAg seroconversion compared to 52% in the chronic inactive hepatitis group ( Wong *et al.*, 2008). Higher pretreatment levels can facilitate viral suppression by NA ( Zeuzem *et al.*, 2009; Wong and Chan, 2009). The role of baseline ALT in virological response is more dominant in people with HBeAg positive status compared to HBeAg negative ( Lim *et al.*, 2007). This study revealed that low levels of the enzyme increased the risk of not achieving HBV DNA suppression in chronic hepatitis B patients with TDF by 1.37 (p=0.037). The multivariate analysis showed that ALT levels were independently associated with the suppression (p=0.036), but there was no relationship between AST levels and suppressed HBV DNA (p=0.234). Although AST is an enzyme produced by hepatocytes, it is also found in the skeletal muscle, heart muscle, and kidney tissue. This indicates that its specificity as a hepatocellular marker is lower than ALT ( Hall and Cash, 2012).

The viral clearance by the host was influenced by the low amount of HBV DNA in the serum. Furthermore, its baseline levels are associated with a higher possibility of HBeAg loss or seroconversion in HBeAg-positive patients ( Zeuzem *et al.*, 2009), while low/undetectable levels are related to a virological response ( Lim *et al.*, 2007; Lok, 2019). The amount of HBV DNA in the serum is a predictor of chronic hepatitis B prognosis, namely cirrhosis, HCC, and death. The baseline levels also have a association with the effectiveness of NA therapy, including histologic grade, suppression of hepatitis DNA to undetectable, normalization of ALT, and HBeAg seroconversion ( Mendy *et al.*, 2010). This study revealed that high levels of HBV DNA increased the risk of not achieving its suppression in chronic hepatitis B patients with TDF by 1.42 times (p=0.017). The multivariate analysis showed that the levels were independently associated with the HBV DNA suppression (p=0.026).

Continuous exposure to high concentrations of HBV DNA, including HBeAg, HBsAg, and HBx causes anergy in T cells due to increased expression of CTLA-4 ( Ye *et al.*, 2015). Peng revealed that HBeAg can increase the expression in T cells. This is closely associated with high HBV DNA levels, but the underlying mechanism is still unclear ( Peng *et al.*, 2011). Serum HBeAg quantification can be a marker to predict HBV DNA in response to antiviral therapy. Measurement of its levels correlated with the level of HBV DNA replication and liver inflammation. HBeAg seroconversion is also associated with decreased viral load, but this result is controversial. Ji *et al.* reported that 87.5% and 75% of patients with a decrease in HBeAg > 2 logs have undetectable HBV DNA and HBeAg seroconversion, respectively ( Ji *et al.*, 2013). Previous study revealed that its levels were more consistently associated with HBeAg seroconversion than that of HBV DNA ( Yang *et al.*, 2016). Furthermore, its quantification during therapy also has a good prediction of HBV DNA suppression with Peg-IFN therapy. A previous study showed that people with HBeAg > 100 PEIU/mL at week 24 had a 96% probability of not achieving a virological response. HBeAg correlates with virological response in terms of levels, and qualitative status. This study revealed that there was no relationship between HBeAg status and HBV DNA suppression in chronic hepatitis B patients with TDF (p=0.456). However, its levels were not evaluated because the test is not a standard for monitoring chronic HBV.

Hepatitis B can be divided into 10 genotypes based on >8% difference in the genome sequence, namely A-J. Their clinical and virological course also differs with geographies. They have been reported to influence treatment reaction to Peg-IFN. Previous studies revealed that genotypes A and B have a better virological response than C and D ( Marcellin *et al.*, 2013; Sonneveld *et al.*, 2012; Sunbul, 2014). HBV is a strong predictor of virological response to Peg-IFN ( Lok, 2019). It also affects the incidence of mutations in the precore and core promoter regions of the HBV genome in some chronic hepatitis B patients. The dominant precore mutation involves changing the G allele at the nucleotide 1896 (Al896) to A allele, which induces a mutant precore stop codon. This mutation prevents translation of the precore protein and completely inhibits HBeAg production. Furthermore, mutations in the promoter core region have been reported, and the most common type involves the substitution of two nucleotides, namely A-T and G-A at nucleotides 1762 and 1764, respectively. Changes in the promoter core region contribute to the loss of HBeAg. These precore and core promoter mutations have been reported to affect the therapeutic response to IFN treatment, but not to NA. These changes majorly occur in genotypes C and D of HBV ( Alfaresi *et al.*, 2010). Several studies showed that HBV genotypes had no effect on the virological response to NA ( Zeuzem *et al.*, 2009). This is in line with this study that they have no association with HBV DNA suppression in chronic hepatitis B patients with TDF (p=0.549).

The progression of fibrosis in the disease depends on the comorbidities and the degree of active inflammation. Assessment of fibrous liver is important to assess the prognosis and urgency of therapy ( Parikh, Ryan and Tsochatzis, 2017). Sustained HBV suppression with NA is closely associated with decreased necroinflammatory activity and improvement/regression of fibrosis ( Marcellin *et al.*, 2013; Papachrysos *et al.*, 2015). The Knodell, Isaac, and METAVIR histologic systems are often used to assess disease activity as well as to evaluate treatment response. The goal of the therapy is to stop the ongoing necroinflammation and prevent fibrosis progression ( Fernández-Rodríguez and Gutiérrez-García, 2014). The stage of fibrosis determines the prognosis and initiation of treatment, but cannot predict treatment response ( Chen *et al.*, 2021; Grizzi and Desmet, 2014). Its degree is also associated with response to pegylated interferon therapy. Hepatitis B patients who were non-responders to pegylated interferon were closely associated with lighter grading and more severe staging. The grading used to describe the intensity of necroinflammatory activity includes the assessment of portal, periportal, and intraacinar inflammatory cell infiltration, as well as various forms of hepatocyte damage and necrosis. Staging is an evaluation of the degree of fibrosis, which is caused by the necroinflammatory process consisting of F0-F4. However, there is no relationship between the degree and NA therapy ( Lim *et al.*, 2007; Bonino *et al.*, 2007). These findings are consistent with previous studies that the fibrosis stage is not associated with HBV DNA suppression in chronic hepatitis B patients with TDF (p=0.347).

Based on a study conducted by Cho on the effect of TDF administration on CTLA-4 level, it was discovered that an immune molecule that can inhibit T-cell function in chronic HBV patients. After 6 months of therapy, there was a significant decrease in its expression. The result showed that TDF has immunomodulatory properties by lowering CTLA-4 level, which causes T-cell anergy. It also has a functional T-cell recovery effect ( Cho *et al.*, 2020). Wongjitrat revealed that CTLA-4 in chronic HBV patients was significantly higher than the healthy controls. It was also reported to interfere with the immune response and cause chronic hepatitis B persistence ( Wongjitrat *et al.*, 2013). Furthermore, it has an association with HBV DNA suppression in patients with TDF. In the bivariate analysis, subjects with high content are 1.6 times more at risk of not achieving suppression (p=0.002), while the risk was increased by 2.28 times (p=0.009) in the multivariate analysis. Level > 52.2 pg/mL can predict virological response to TDF with sensitivity, specificity, PPV, NPV, and accuracy of 60.4%, 62.6%, 61.8%, 61.3%, and 61.5%, respectively.

In this study, the Hardy-Weinberg test was carried out. The allele and genotype frequencies in the CTLA-4 -1661G>A polymorphism of the subjects were in accordance with the Hardy-Weinberg balance ( Kim *et al.*, 2013; Sha and Zhang, 2011). The results showed that the polymorphism can affect the circulating level. There were significant differences in serum CTLA-4 level between genotypes of the CTLA-4 -1661G>A. The level obtained in chronic hepatitis B patients with genotype GG were significantly higher compared to AG and AA (p<0.001). Furthermore, its amount in the serum were significantly higher in people with the G allele compared to A (p<0.001). The G allele of CTLA-4 -1661G>A showed higher promoter activity than the A allele. The polymorphism was located in the promoter region, which is important for the activation of the transcriptional process. Nucleotide sequence analysis showed binding sites with potential transcription factors, namely nuclear factor of activated T-cells (NFAT), Activator Protein 1 (AP-1), signal transducer and activator of transcription (STAT) ( Guo *et al.*, 2020). The allele variation of the CTLA-4-1661G>A gene can increase the transcription factors, including NFAT, AP-1, and STAT, which bind to the promoter site. This is the initial site for the attachment of the RNA polymerase enzyme, which perform transcription. Furthermore, transcription factors are proteins that control the rate of genetic information transcribed. These factors or their combination with other proteins in a complex can activate RNA polymerase recruitment and binding stabilization, as well as catalyze histone acetyltransferase (HAT) activity. This then causes an increase in the transcription rate and expression of CTLA-4 [9, 15,67]. The factor Foxp3 in the promoter region of CTLA-4 can recruit histone acetyltransferase enzymes, which stimulate histone acetylation processes. It can also regulate transcription through direct chromatin remodeling ( Kumar *et al.*, 2014). The regulation of CTLA-4 cell surface expression can be explained by the functional variations in the amino acid sequence of the molecule located at the cytoplasmic tail Y201 motif. Furthermore, this motif is an important intracellular regulator of CTLA-4 protein level through its interaction with the clathrin adapter complexes AP-1 and AP-2, which mediate, degrade and endocytose the protein, respectively. The newly synthesized CTLA-4 does not remain in the Golgi or post-Golgi compartment. It is also not stored in cytoplasmic vesicles, but rather transported directly to the cell surface ( Schneider and Rudd, 2014).

There is an association between CTLA-4 -1661G>A gene polymorphism with HBV DNA suppression in chronic hepatitis B patients with TDF. People with GG+AG genotype are 1.42 times more at risk of not achieving suppression (p=0.031), while patients with the G allele of CTLA-4 -1661G>A have a 1.27-fold increased risk compared to others with the A allele (p=0.025). Based on the multivariate analysis, people with genotype GG+AG are 1.52 times more at risk of not achieving HBV DNA suppression (p=0.041). The effect of the CTLA-4-1661G>A gene polymorphism on virological response to TDF is caused by an increase in CTLA-4 level. Furthermore, CTLA-4 molecules through their interactions with costimulatory molecules B7 can cause negative regulation of T cells. Their increased expression can also increase spontaneous T cell apoptosis. CTLA-4 can influence infection progression and antiviral immune response ( Tavakolpour, Alavian and Sali, 2016). Treatment with NA enhances specific CD8+ T-cell function and improves anergy by up-regulating CTLA-4 ( Thimme and Dandri, 2013).

This study demonstrated a dose-dependent effect of the G allele in increasing serum CTLA-4 level as well as the risk of not achieving a virological response to TDF. Moreover, CTLA-4 production and risk of non-responders to TDF were high in patients with the GG genotype, intermediate in AG, and low in AA, while the A allele was protective.

This study had limitations, including not analyzing quantitative HBeAg, quantitative HBsAg, and polymorphisms of other genes that can affect the virological response to TDF. This study also only analyzed 1 location of the CTLA-4 gene, namely -1661G>A.

The CTLA-4 -1661G>A polymorphism can be used as a predictor of chronic hepatitis B treatment response so that appropriate management and ongoing monitoring can be done to achieve treatment targets and prevent the progression of chronic hepatitis B. Further research is needed to determine the role of anti-CTLA-4 in increasing the virological response to TDF.

## Conclusion

The results showed the factors affecting HBV DNA suppression in chronic hepatitis B patients with tenofovir disoproxil fumarate (TDF) were the levels of CTLA-4, HBV DNA, ALT, and CTLA-4-1661G>A polymorphism. There was no relationship between gender, age, ethnicity, obesity, baseline AST, HBeAg, genotype, liver fibrosis and HBV DNA suppression after 24 weeks of treatment (p>0.05).

## Data Availability

Figshare: Mater Data Hepatitis. https://doi.org/10.6084/m9.figshare.21471264 ( Darmadi, 2022a). This project contains the following underlying data:
- Master Data.xlsx (Data consists of polymorphisms towards HBV DNA) Figshare: The result of CTLA4 1661GA and CTLA ELISA https://doi.org/10.6084/m9.figshare.21651587 ( Darmadi, 2022b). This project contains the following underlying data:
- Hasil CTLA4 1661GA dan CTLA4 ELISA.csv (This is the result of CTLA4 1661GA and CTLA ELISA) - RT-PCR CTLA4 1661GA (standard curves from RT-PCR) Figshare: Informed consent for sample https://doi.org/10.6084/m9.figshare.21564474 ( Darmadi, 2022c). Data are available under the terms of the Creative Commons Attribution 4.0 International license (CC-BY 4.0).
