# Supplementary material for: Factors affecting HBV DNA suppression in chronic hepatitis B patients treated with tenofovir disoproxil fumarate
Source: F1000Res. 2023 Aug 24;11:1521. Originally published 2022 Dec 15. [Version 2] doi: 10.12688/f1000research.128116.2 (PMC10521109; doi:10.12688/f1000research.128116.2)
Supplement: Peer review history

# Reviewer report: Reviewer response for version 2

**DOI:** 10.5256/f1000research.154811.r199607

**Published:** 2023-09-14

**Authors:** I Ketut Mariadi

**Affiliations:** 1 Gastroenterohepatology Division, Department of Internal Medicine, Prof. Ngoerah General Hospitalglah General Hospital, Udayana University, Denpasar, Indonesia

This revised article accommodates my suggestions. 

 I have no further comment.

Is the work clearly and accurately presented and does it cite the current literature?

Partly

If applicable, is the statistical analysis and its interpretation appropriate?

Partly

Are all the source data underlying the results available to ensure full reproducibility?

Yes

Is the study design appropriate and is the work technically sound?

Partly

Are the conclusions drawn adequately supported by the results?

Yes

Are sufficient details of methods and analysis provided to allow replication by others?

Yes

Reviewer Expertise:

Hepatology and gastroenterology

I confirm that I have read this submission and believe that I have an appropriate level of expertise to confirm that it is of an acceptable scientific standard.

# Reviewer report: Reviewer response for version 2

**DOI:** 10.5256/f1000research.154811.r199608

**Published:** 2023-09-14

**Authors:** Hendra Koncoro

**Affiliations:** 1 Department of Internal Medicine, Sint Carolus Hospital, Jakarta, Indonesia

I have read the revised article and approve to the revised article without any further revision.

Is the work clearly and accurately presented and does it cite the current literature?

Partly

If applicable, is the statistical analysis and its interpretation appropriate?

Partly

Are all the source data underlying the results available to ensure full reproducibility?

Yes

Is the study design appropriate and is the work technically sound?

Yes

Are the conclusions drawn adequately supported by the results?

Yes

Are sufficient details of methods and analysis provided to allow replication by others?

Yes

Reviewer Expertise:

NA

I confirm that I have read this submission and believe that I have an appropriate level of expertise to confirm that it is of an acceptable scientific standard.

# Reviewer report: Reviewer response for version 1

**DOI:** 10.5256/f1000research.140677.r174440

**Published:** 2023-05-30

**Authors:** Hendra Koncoro

**Affiliations:** 1 Department of Internal Medicine, Sint Carolus Hospital, Jakarta, Indonesia

Darmadi et al have done a very good and useful study to find out factors affecting HBV DNA suppression in chronic hepatitis B treated with Tenofovir Disoproxil Fumarate (TDF). TDF is one of the first-line drugs used for treatment of chronic hepatitis B. Previous studies have shown longterm nucleotide analog therapy (eg: TDF) can increase peripheral T cell levels to enhance antiviral response. Their activation is strongly determined by genetic factors, such as CTLA-4. It's really interesting that Darmadi et al focused on the association of CTLA-4 polymorphism in the persistence and progression of chronic hepatitis B infection, with HBV DNA suppression. Although this study has so many good points, there might be some suggestions and comments to improve this research article.

 First of all, there is a similarity in the abstract at the results and conclusion section. Preferably, the results are presented with numerical data.

 Then, in the methods, it is stated that "the case group was patients who did not achieve virological response to TDF treatment, the control group was patients who achieved a virological response to TDF treatment." The author need to give definition of virological response to NUCs (eg. Tenofovir). Inclusion criteria was also did not mention in the Methods section.

 In the procedure section, CTLA-4 level was measured, but the author did not state when the measurement was done. The author should state whether the measurement was done before, after, or before and after the treatment started.

 In the data analysis, it is important to explain the cut off and how to define each of the categorical variables, eg. the age is divided into old and young, the author need to state the criteria of old and young. The AST and ALT baseline is better categorized as normal and increased instead of low and high.

 In the discussion section, the author has written down the basic theory and supporting studies that are advanced and complete. However, it is better to use the latest reference to optimize and support this study as there are several references dates up to 24 years ago. In the discussion it should be stated how this research may change the daily clinical practice of clinicians. Does CTLA-4 levels or CTLA-4 polymorphism need to be checked for every chronic hepatitis B patients naive-treatment to achieve ideal or satisfactory end point or just to measure HBV DNA suppression.

Is the work clearly and accurately presented and does it cite the current literature?

Partly

If applicable, is the statistical analysis and its interpretation appropriate?

Partly

Are all the source data underlying the results available to ensure full reproducibility?

Yes

Is the study design appropriate and is the work technically sound?

Yes

Are the conclusions drawn adequately supported by the results?

Yes

Are sufficient details of methods and analysis provided to allow replication by others?

Yes

Reviewer Expertise:

Hepatitis B, Liver Cirrhosis, Hepatocellular Carcinoma

I confirm that I have read this submission and believe that I have an appropriate level of expertise to confirm that it is of an acceptable scientific standard, however I have significant reservations, as outlined above.

# Reviewer report: Reviewer response for version 1

**DOI:** 10.5256/f1000research.140677.r168445

**Published:** 2023-04-21

**Authors:** I Ketut Mariadi

**Affiliations:** 1 Gastroenterohepatology Division, Department of Internal Medicine, Prof. Ngoerah General Hospitalglah General Hospital, Udayana University, Denpasar, Indonesia

I think this paper is excellent and is an important addition to the literature. The new idea in determining the response to hepatitis B treatment is important. but I have some suggestions for this paper:

1. The results of the study in the abstract should be mentioned clearly with the data, not the same as the conclusions.
2. Some of the reference literature is older than 10 years. 
3. The aim of the study, the results, and the conclusion should be in line.
4. Please use the term relation, correlation, and association properly
5. Describe the study design clearly. This is a case-control study, how to find and determine the case and control to reduce selection bias. how to find 91 cases and 91 control out of 276 samples ( after doing exclusion ). And not clear when you did the test of CTLA-4, at the start of treatment or at the end of treatment?
6. Please state clearly, how to determine categorical variables from the numerical variable. ex: High vs Low in AST, ALT, DNA etc. or high vs normal?
7. Table 5 should be presented clearly
8. Please make a clear statement in the text to avoid misinterpretations. ex. in the results: "There was a significant relationship between low baseline ALT, high baseline HBV DNA, high CTLA-4 levels, and HBV DNA suppression (p<0.05) (Darmadi, 2022a, 2022b)." " low baseline ALT are 1.37 times more at risk of not achieving HBV DNA suppression (p=0.037)," 

Is the work clearly and accurately presented and does it cite the current literature?

Partly

If applicable, is the statistical analysis and its interpretation appropriate?

Partly

Are all the source data underlying the results available to ensure full reproducibility?

Yes

Is the study design appropriate and is the work technically sound?

Partly

Are the conclusions drawn adequately supported by the results?

Yes

Are sufficient details of methods and analysis provided to allow replication by others?

Yes

Reviewer Expertise:

Hepatology and gastroenterology

I confirm that I have read this submission and believe that I have an appropriate level of expertise to confirm that it is of an acceptable scientific standard, however I have significant reservations, as outlined above.
